# Arsenical Association: Inorganic Arsenic May Accumulate in the Meat of Treated Chickens

**DOI:** 10.1289/ehp.121-a226

**Published:** 2013-07-01

**Authors:** Charles W. Schmidt

**Affiliations:** Charles W. Schmidt, MS, an award-winning science writer from Portland, ME, has written for *Discover Magazine*, *Science*, and *Nature Medicine*.

Until recently, chicken producers would routinely supplement poultry feed with a growth-promoting arsenical drug called roxarsone, which also treats intestinal parasites in the birds and gives their meat an appealing pink color. A study in this issue of *EHP* shows that inorganic arsenic (iAs) accumulates in the breast meat of broiler chickens, potentially as a result of treatment with roxarsone.^1^

In 2011 the U.S. Food and Drug Administration (FDA) found that the livers of roxarsone-treated chickens had elevated levels of iAs,^2^ a known human carcinogen.^3^ In response, roxarsone’s manufacturer, Pfizer, voluntarily pulled the drug off the U.S. market, although it is still sold overseas, and a similar arsenical drug is still available in the United States.^4^ Sampling for the new study took place between December 2010 and June 2011, before Pfizer withdrew roxarsone from the U.S. market.

Roxarsone is an organic form of arsenic, which although less toxic to humans than the inorganic species implicated in cancer, has been shown to affect the growth of endothelial cells in culture.^5^ When roxarsone was approved by the FDA it was believed the drug passed through chickens unchanged. The FDA and new *EHP* studies each suggest that roxarsone can transform into iAs and accumulate in the edible portions of the birds, making the toxic metal available for human consumption.

For the current study, lead author Keeve Nachman, director of the Farming for the Future program at the Johns Hopkins Center for a Livable Future, and colleagues analyzed chicken breast meat samples from three categories: 1) conventional chickens for which arsenical drug use was permitted (69 samples); 2) conventional antibiotic-free chickens for which arsenical drug use was unlikely but possible since arsenical drugs are not considered antibiotics (34 samples); and 3) chickens certified as organic by the U.S. Department of Agriculture, which are not fed roxarsone and other arsenical feed additives (37 samples). The samples came from 82 stores in 10 U.S. metropolitan areas. Some of the samples underwent arsenic speciation, and for a subset of these the authors compared paired cooked and raw samples.

The results showed that iAs was highest in cooked conventional chicken meat (with a geometric mean of 1.8 µg/kg) and lowest in cooked organic chicken meat (with a geometric mean of 0.6 µg/kg). According to Nachman, arsenic found in organic chicken meat reflects exposure from other potential sources, such as drinking water. In addition, chicken meat with detectable roxarsone had higher iAs concentrations than chicken meat without detectable roxarsone.^1^

**Figure:**
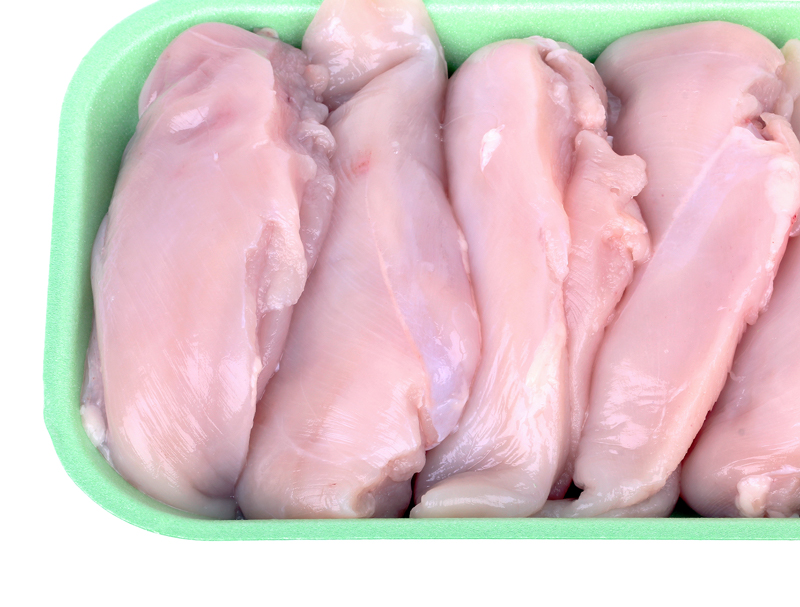
Inorganic arsenic detected in chicken meat could reflect dosing of birds with arsenical drugs. © photopixel/Shutterstock

The study authors also performed a risk analysis based on iAs concentrations measured in their samples, estimates of the amount of chicken consumed over a 70-year lifetime, and the relationship between iAs consumption and the risk of cancer. Based on these assessments, they estimated that if all chicken producers were to use arsenic-based drugs, the added iAs exposure from consuming chicken would result in an additional 3.7 bladder and/or lung cancers per 100,000 people, or an average of 124 cancers in the United States per year.^1^ “Our study gives the FDA a clear rationale for withdrawing its approval for roxarsone and potentially other arsenic-based drugs in animal agriculture,” Nachman says.

The authors’ risk assessment assumed a cancer slope factor for iAs that’s more than 17 times higher than the cancer slope factor adopted by the EPA in 1998 for skin cancer.^6^ According to Yu Chen, an associate professor at the New York University School of Medicine, the higher value reflects epidemiological evidence from studies conducted in Taiwan suggesting heightened risks for bladder and lung cancer from iAs exposure. But the higher cancer slope factor—which was proposed by the EPA in 2010 and has since been withdrawn pending further agency review—has been heavily disparaged by industry stakeholders, who criticize the methods and data used by the EPA in its dose–response assessment.^7^ A National Research Council panel is currently evaluating the EPA’s toxicological assessment of iAs.

In the meantime, Amy Sapkota, an assistant professor at the University of Maryland School of Public Health who was not involved in the study, argues that the study is strong. She says, “It provides FDA with good data about whether it should formally withdraw the use of arsenicals from chicken production in the U.S.”
