# Erratum: “Arsenical Association: Inorganic Arsenic May Accumulate in the Meat of Treated Chickens”

**DOI:** 10.1289/ehp.121-a241

**Published:** 2013-08-01

The July 2013 News article “Arsenical Association: Inorganic Arsenic May Accumulate in the Meat of Treated Chickens” [Environ Health Perspect 121:A226 (2013)] contained a typographical error. Milligrams should have been expressed as micrograms.

*EHP* regrets the error.

